# How consistent are sick leave assessments? Variation among primary care physicians in Sweden

**DOI:** 10.1080/02813432.2025.2577665

**Published:** 2025-10-29

**Authors:** Cecilia Rosander, Hanna Israelsson Larsen, Elin Karlsson, Jon Pallon, Maria Samefors, Hans Thulesius, Magnus Falk

**Affiliations:** a Department of Health, Medicine and Caring Sciences, Linköping University, Linköping, Sweden; b Kärna Primary Care Centre, Linköping, Sweden; c Cityhälsan Centrum Primary Care Centre, Norrköping, Sweden; d Department of Clinical Sciences in Malmö, Family Medicine, Lund University, Malmö, Sweden; e Department of Research and Development, Region Kronoberg, Växjö, Sweden; f Rosenhälsan Primary Care Centre, Huskvarna, Sweden; g Department of Medicine and Optometry, Linnaeus University, Kalmar, Sweden

**Keywords:** Work capacity, sick leave, primary care physicians, assessment, sick days

## Abstract

**Introduction:**

Assessing work capacity and determining appropriate sick leave is a complex process. Despite the importance of fair and consistent assessments, evidence suggests that evaluations may vary. However, previous studies have been inconclusive and have mainly focused on whether sick leave should be recommended or not. The present study examined the medical reliability and consistency of physicians’ assessments of work capacity, as indicated by the reported percentage of reduced work capacity and the recommended length of sick leave.

**Methods:**

A cross-sectional survey was conducted with 142 primary care physicians from four Swedish counties. Participants assessed six anonymised, authentic medical certificates related to mental health and pain-related conditions. For each case, they estimated the degree of reduced work capacity and the recommended sick leave duration, which were combined into a sick leave score. Physician characteristics (e.g. gender, professional status) and perceived managerial support were also measured.

**Results:**

There was considerable variation in sick leave scores, both across physicians and between certificates. Male physicians and those with more years of experience recommended longer sick leave for pain-related cases, while specialists showed less variation in their assessments. Perceived support from frontline managers was associated with lower scores. No significant associations were found for patient involvement.

**Conclusions:**

The findings suggest that sick leave assessments may lack consistency and be influenced by physician-related factors, particularly for pain-related conditions. Stronger support structures, opportunities for knowledge exchange, and clearer guidelines may help reduce unwarranted variation and promote more reliable assessments.

## Introduction

High levels of sick leave are a concern in many European countries [1]. Although sickness insurance systems vary, all Western European countries require a medical certificate in which a physician verifies the individual’s inability to work normally [2]. During this process, the physician must assess whether the patient is capable of performing their job despite activity limitations, or whether their ability to work is fully or partially impaired [3]. Many primary care physicians find sickness certification challenging [4]. Contributing factors include limited time, insufficient interprofessional collaboration, and inadequate training in insurance medicine – all of which may impair physicians’ ability to make well-founded assessments of reduced work capacity [5,6]. Difficulties may also arise from limited knowledge of the patient’s workplace conditions, making it hard to translate functional impairments into concrete judgements about work ability [6]. Further complicating the process are physicians’ dual roles as treating clinicians and as medical experts providing information to employers or the Social Insurance Agency [7–10]. Additional challenges may arise when assessing patients with conditions lacking clear objective findings. In such cases, physicians often rely on tacit knowledge and their clinical experience [6,11,12]. These assessments may also be shaped by the physician’s professional approach, personality, gender, and organisational context, and are frequently patient-driven – that is, based largely on the patient’s own account of their limitations [13–16].

Previous studies have examined variation in physicians’ assessments of work capacity and the need for sick leave. There are indications of such variation, for example depending on whether the physician was a man or a woman, but the results are inconclusive [13,17], and there is still insufficient knowledge about which factors influence assessments related to the duration of sick leave. One possible explanation is that many of these studies have relied on vignette studies, that is, studies using fictional patient cases [14,17,18]. Some studies have used actual cases, but typically the outcome has only concerned whether sick leave was granted or not [19], an approach that is also common in vignette studies. However, there are no studies in which assessments of the degree of reduced work capacity, in terms of both the percentage and duration of sick leave, have been explored using real patient cases rather than fictional scenarios.

Some studies have used actual cases, but typically the outcome has only concerned whether sick leave was granted or not [19], an approach that is also common in vignette studies. However, there are no studies in which assessments of the degree of reduced work capacity, in terms of both the percentage and duration of sick leave, have been explored using real patient cases rather than fictional scenarios.

Sick leave has been described as comparable to a medical treatment – it may produce both intended effects and adverse consequences and therefore requires careful consideration in terms of both dosage and duration [20]. Too short a period away from work may lead to relapse, while excessively prolonged sick leave has been associated with negative outcomes, including more persistent depressive symptoms [21]. If patients are to receive appropriate decisions, there must be reliability in these assessments. However, there is a risk that factors not directly related to a specific patient’s case may influence the outcome.

### The challenges of assessing work capacity

Work capacity can be defined as the patient’s resources and abilities in relation to the demands of their job [22]. In addition to individual factors, work capacity is influenced by job tasks, the work environment, and the person’s competence and motivation [22]. To facilitate return to work, it is desirable that assessments of work capacity focus on the patient’s resources and opportunities [23]. Reported challenges in such assessments include identifying the specific demands of the patient’s job, understanding how the work environment affects the individual, and determining how these factors interact with the patient’s capacity to work [11]. Importantly, such assessments should not only identify potential risks but also provide a basis for considering what workplace modifications may be needed and feasible to support return to work. In many cases, the assessment relies on the patient’s own description of their symptoms in relation to their work tasks. Although physicians have extensive knowledge of illness, they express uncertainty about which non-medical factors should be considered when assessing work capacity [6]. There is also a lack of clear guidelines on how such knowledge should be applied in the assessment of reduced work capacity in individual cases [3].

Alexanderson et al. [4] found a growing perception among physicians that managing sick leave cases is problematic, with primary care physicians reporting the greatest challenges. In primary care, the most difficult aspects are reported to be diagnosing mental health conditions and evaluating the extent to which a patient’s health problems affect their ability to function and perform work-related activities [5,24].

Many physicians also report difficulties when there is disagreement with the patient regarding the need for a medical certificate or sick leave. Such disagreements may result in unnecessarily long periods of sick leave [8,9,25]. Moreover, medical certificates issued while waiting for action from the employer, the healthcare system, or the Swedish Social Insurance Agency can also contribute to prolonged sick leave [26,27]. These extended periods may, in turn, lead to further deterioration in the patient’s health and financial situation [28]. It has been shown that establishing shared routines and clear workplace policies reduces the risk of excessively long periods of sick leave [27]. The length of the sick leave period also affects how problematic physicians perceive the management of sick leave cases to be. Alexanderson et al. [4] showed that longer sick leave cases are perceived as particularly challenging.

Another common challenge for physicians is deciding whether to extend a sick leave period initially assessed by another physician [24,26]. Physicians report a lack of time during consultations and insufficient routines for work involving insurance medicine [5]. More than half (53%) of all primary care physicians report difficulties in assessing patients’ functional limitations [5,26], and 65% find it difficult to evaluate activity limitations in relation to the patient’s job [5]. Many physicians describe a lack of basic conditions – such as time constraints, poor interprofessional collaboration, and inadequate training in insurance medicine – which limits their ability to make accurate assessments of reduced work capacity [5,6]. In addition, limited knowledge of workplace factors makes it difficult to translate functional limitations into assessments of work capacity [6].

### Context of the study

In Sweden, individuals can self-certify sickness absence for up to seven days. From day eight onwards, a medical certificate issued by a physician is required, explaining how the illness, functional impairments, and activity limitations affect the person’s work capacity. From day 15 onwards, this documentation provides the basis for the Swedish Social Insurance Agency to determine eligibility for benefits or support [29]. Physicians assess whether the individual’s work capacity is reduced by 25%, 50%, 75%, or 100%. During the first 14 days, the employer provides sick pay. From day 15, the individual may apply for sickness benefits from the Swedish Social Insurance Agency [30].

In a Western European comparison, sickness absence is influenced by how sickness insurance systems are structured [2]. In Sweden and Norway, sickness absence is primarily covered by the mandatory public social insurance system, and employees enjoy strong employment protection. In contrast, in the United Kingdom and the Netherlands, the employer is responsible for providing compensation during sickness absence, and in Denmark and the UK, employment protection is generally weaker. In Germany and France, employees are insured through their employer [2].

Employers in Sweden, Norway, Finland, Denmark, Germany, France, and the UK are entitled to request a medical certificate confirming the employee’s incapacity to work. In the Netherlands, the employee reports their sickness to the employer and is then referred to a physician employed by the company. This physician provides information to the employer regarding the condition, the degree of incapacity, and the expected timeline for return to work [2]. Despite the structural differences in sickness insurance systems, all Western European countries require a medical certificate to verify an individual’s incapacity to work [2].

### Aim and research questions

The aim of the present study was to examine the medical reliability and consistency of physicians’ assessments of work capacity, as indicated by the reported percentage of reduced work capacity and the recommended length of sick leave. Considerable variation in these assessments could have implications for patient safety and the fair allocation of sickness benefits. The study also explored factors that may influence how physicians assess patients’ reduced work capacity. This aim was explored through the following research questions (RQs):

RQ1: How do assessments of reduced work capacity vary between primary care physicians, based on (a) individual certificates and on (b) aggregated assessments of pain-related and mental health-related cases?

RQ2: Are there differences in the *variability* of estimated reduced work capacity between physicians based on gender, competence, patient involvement, and time since graduation, when assessing certificates related to (a) pain conditions and (b) mental health conditions?

RQ3: Are there differences in the *average* estimated reduced work capacity between physicians based on gender, competence, patient involvement, and time since graduation, when assessing certificates related to (a) pain conditions and (b) mental health conditions?

RQ4: Are there associations between the estimated reduced work capacity and (a) physicians’ characteristics and (b) their perceived work support?

## Materials and methods

### Design and sample

The present cross-sectional study is based on data from 16 primary healthcare centres across four counties in Sweden, collected through primary care physicians’ responses to a designated study survey. An invitation to participate in the study was sent to the managers and the medically responsible physicians at 40 healthcare centres within the four regions. Initially, we sent the invitation to all healthcare centres in one county and later expanded it to include selected healthcare centres in three neighbouring counties. Efforts were made to ensure a mix of rural and urban locations, as well as both public and private healthcare providers. All those who responded to the invitation were contacted to schedule an appointment for data collection at each healthcare centre, which took place during the autumn of 2024. The physicians who were present at these appointments were provided with necessary information about the study and gave their informed consent to participate. Everyone who was present chose to participate.

### The survey

Data were collected using a survey primarily focused on the assessment of six anonymised, authentic medical certificates related to diagnoses including mental health conditions and/or pain-related issues. These diagnoses were selected because they represent the most common causes of sick leave among both women and men in Sweden [31]. The selection of the medical certificates to be used in the study was conducted in two steps. First, a total of 15 patients who had been on sick leave due to a mental health condition or a musculoskeletal pain-related issue during the spring of 2024 were identified with the assistance of the rehabilitation coordinators at two primary care centres. The physicians who had issued these medical certificates were contacted and asked to provide consent for their respective patients to be approached. In the second step, the patients to whom the certificates referred were contacted. Initially, patients were contacted by phone and received verbal information about the study. Written information, along with a consent form, was then sent to their homes. This allowed the patients to review the study details before deciding whether to permit the use of their medical certificate in the study. Twelve patients returned written consent. From this group, six medical certificates were selected, as this was considered the maximum number each participating physician could reasonably be expected to assess. The main selection criteria were that the certificates were representative and included a sufficient level of complexity and variation in severity. Certificates 1 and 6 addressed mental health conditions, whereas certificates 2, 4, and 5 addressed pain-related issues. Certificate 3 included both mental health and pain as documented reasons for sick leave and was not included in either of the two certificate groups.

All six certificates were included in the survey after removal of the original certifying physician’s assessment of reduced work capacity and prognosis for return to work. Based on the information provided in each certificate, including diagnosis, functional impairments, activity limitations, medical treatment, and any additional notes, participating physicians were asked to make their own assessments of the initial percentage reduction in work capacity (0, 25, 50, 75, or 100%) and the number of weeks of sick leave at that level.

Additionally, data were collected on the participating physicians’ gender, professional status, years of professional experience, patient involvement, and perceived support from their frontline manager in handling sick leave cases. Professional status was assessed using a question with response options indicating whether the physician was a specialist, resident physician, intern, or junior physician. Patient involvement was assessed using three items measuring different aspects of how much the physician typically involves the patient: ‘I always strive to involve the patient in my assessment of reduced work capacity’, ‘I always strive to involve the patient in my assessment of return to work’ and ‘The patient and I jointly set goals for return to work’. Perceived support from the frontline manager was assessed using the item: ‘I receive strong support from my frontline manager in handling sick leave cases’. The responses for the items capturing patient involvement and frontline manager support were given on a seven-point Likert scale.

The content of the survey and the medical certificates was discussed within the research team. To ensure clarity, the survey was then pilot tested on a small group of primary care physicians who were not affiliated with the research project. This pilot group included physicians with varying professional statuses. Following this initial pilot study, minor revisions were made to the survey protocol.

### Procedure

All participating healthcare centres were visited by the first author during scheduled staff meetings or similar gatherings to facilitate data collection. This approach ensured that instructions were delivered as consistently as possible and that any questions could be addressed directly. The time required to complete the survey for those participating on site ranged from 35 to 45 min. The survey was available in both paper and digital formats, allowing physicians who were not present during the visits to participate in the study.

### Study participants

The study is based on data from 142 primary care physicians; 137 participated on site, while five responded digitally (see Table 1 for sample characteristics). The gender distribution was relatively balanced, with a slight majority of women. Notably, employment duration varied considerably, ranging from a few months to 50 years. Similarly, both years since graduation and participants’ ages showed substantial variation, ranging from 1 to 53 years since graduation and from 25 to 82 years of age.

**Table 1.** Characteristics of the participating physicians.

	*N*	%
Gender		
Male	60	42%
Female	82	58%
Professional status		
Specialist	85	60%
Non-specialist	57	40%
Resident physician	38	27%
Intern/Junior physician	19	13%
Country of graduation		
Sweden	118	83%
Outside Sweden	8	6%
Not reported	16	11%
	Mean (*SD*)	Median
Age	41.8 (11.3)	40.0
Employment duration	9.9 (9.7)	7.0
Years since graduation	14.3 (10.8)	12.0

### Data analysis

All analyses were conducted using SPSS version 29. To obtain a comparable measure of reduced work capacity, a sick leave score was calculated by multiplying the number of weeks of estimated sick leave by the percentage of reduction in work capacity. On their own, neither of these measures is comparable between physicians, as they are interdependent (e.g. six weeks of recommended sick leave at 25% is not comparable to two weeks at 100%). The sick leave score reflects the total extent of recommended absence from work, expressed as the equivalent of full-time sick leave in weeks. This score was calculated for each individual certificate, as well as for the group of certificates related to mental health conditions and to pain. It is important to note that the sick leave score does not refer to the patient’s actual absence from work, but to the estimate recommended by each participating physician. All main results were based on this score.

In the analyses, specialists were compared with non-specialists. Years of professional experience were measured as the number of years since graduation and were also dichotomised into short versus long time since graduation using a median split (Md = 12 years). For patient involvement, a mean score was calculated across the three items, with a Cronbach’s alpha of .80, indicating good internal consistency. This variable was also dichotomised using a median split (Md = 5.7).

Descriptive statistics were first examined for participants’ total estimated sick leave duration for each medical certificate, as well as for the aggregated measures based on the two certificates concerning mental health conditions and the three concerning pain-related cases. To estimate overall confidence intervals for sick leave score, bootstrapping with 1000 samples was applied (95% bootstrap confidence intervals, 95% BootCI). Intercorrelations were calculated using Pearson’s r.

To investigate differences in variability across the predefined groupings, Levene’s test was used. A significant result indicated a difference in variance (RQ2). To address RQ3, differences in mean total sick leave duration were analysed using t-tests. In cases of unequal variance, Welch’s test was applied to adjust for the difference.

## Results

**Figure 1.**
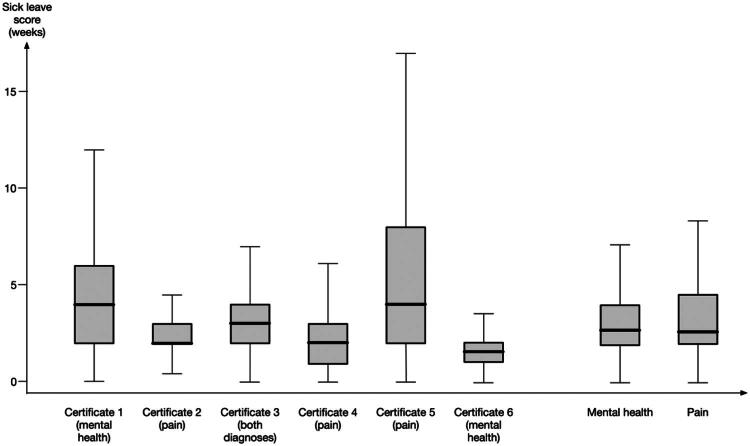
Boxplot of sick leave score across individual medical certificates and aggregated diagnostic groups, showing medians, interquartile ranges (IQR) and 1.5 x IQR.

To address RQ1, we examined the overall variation in the sick leave score across all medical certificates and diagnostic groups. There was considerable variation in sick leave score, ranging from 0 to 52 weeks for the same certificate. Substantial differences were also observed in mean values, with 95% confidence intervals spanning several weeks. These variations in physicians’ assessments of reduced work capacity, expressed as the sick leave score, across cases and diagnostic groups (RQ1) are presented in Table 2, which presents descriptive statistics for the six medical certificates, as well as for the aggregated measures for mental health conditions (certificates 1 and 6) and pain (certificates 2, 4, and 5). Figure 1 displays separate boxplots for the individual certificates and the aggregated measures.

**Table 2.** Descriptive statistics for sick leave score across individual medical certificates and aggregated diagnostic groups.

Sick leave score^a^	Mean	*SD*	95% BootCI	Min	25th perc.	Median	75th perc.	Max
Certificate 1 (mental health)	4.56	3.61	[4.00; 5.19]	1	2.00	4.00	6.00	26
Certificate 2 (pain)	2.56	1.64	[2.32; 2.84]	0	2.00	2.00	3.00	13
Certificate 3 (both diagnoses)	3.55	3.75	[2.99; 4.22]	0	2.00	3.00	4.00	36
Certificate 4 (pain)	2.64	4.22	[2.04; 3.37]	0	0.94	2.00	3.00	36
Certificate 5 (pain)	5.99	7.04	[4.94; 7.30]	0	2.00	4.00	8.00	52
Certificate 6 (mental health)	1.80	2.10	[1.49; 2.18]	0	1.00	1.50	2.00	20
Mental health certificates	3.18	2.55	[2.81; 3.61]	0.25	1.94	2.75	4.00	22.75
Pain certificates	3.63	3.47	[3.12; 4.31]	0	2.00	2.67	4.54	29.33

^a^ Calculated by multiplying the number of weeks of estimated sick leave by the percentage of reduced work capacity.

### Differences in variability and mean sick leave score

Group differences in the variation (RQ2) and mean values (RQ3) of the sick leave score were analysed based on physician gender, professional status, patient involvement, and time since graduation. Separate analyses were conducted for certificates related to mental health conditions and those related to pain. Table 3 presents the results, including tests of variability and mean differences.

**Table 3.** Means, standard deviation, Levene’s test and t-test for the sick leave score for mental health and pain issues for gender, competence, patient involvement and time since graduation.

				Differences in variance		Differences in mean	
				(Levene’s test)		(t-test)	
Grouping	Issue	Mean	*SD*	*F*	*p*	*t (df)*	*p*
Male physician	Mental health	3.53	**3.51**	8.15	.005	1.27 (74.18)	.207
Female physician		2.92	**1.47**				
Male physician	Pain	**4.40**	**4.77**	8.64	.004	2.02 (73.02)	.047
Female physician		**3.08**	**1.95**				
Male physician	Total	4.08	**3.79**	14.12	<.001	1.99 (71.85)	.051
Female physician		3.06	**3.06**				
Specialist	Mental health	3.04	**1.49**	9.28	.003	−0.70 (69.42)	.486
Non-specialist		3.39	**3.59**				
Specialist	Pain	3.69	3.82	0.09	.767	0.24 (140)	.814
Non-specialist		3.55	2.91				
Specialist	Total	3.45	2.24	2.44	.121	−0.22 (140)	.826
Non-specialist		3.55	3.36				
Patient involvement	Mental health	3.05	2.95	0.45	.502	−0.58 (139)	.563
Patient uninvolved		3.30	2.05				
Patient involvement	Pain	3.79	4.34	2.70	.103	0.55 (139)	.595
Patient uninvolved		3.48	2.24				
Patient involvement	Total	3.44	1.99	2.38	.125	0.20 (139)	.840
Patient uninvolved		3.54	3.31				
Short time since graduation	Mental health	2.91	1.96	0.37	.394	−2.89 (136)	.373
Long time since graduation		3.21	1.97				
Short time since graduation	Pain	**2.92**	**1.80**	5.54	.020	−2.28 (79.22)	.025
Long time since graduation		**4.28**	**4.41**				
Short time since graduation	Total	**2.96**	1.75	1.01	.317	−2.27 (136)	.025
Long time since graduation		**3.83**	2.70				

*Notes:* In cases of unequal variance, a Welch test was used to test the mean difference. Significant differences are marked in bold text for clarity.

Regarding variability (RQ2), a significant difference in variance was found between male and female physicians in their assessments of patients, as reflected in the sick leave score for both mental health conditions (*p* = .005) and pain-related conditions (*p* = .004), with male physicians displaying greater variability in both cases. A similar pattern was observed for mental health conditions when comparing specialists and non-specialists, with non-specialists showing significantly greater variation in their assessments (*p* = .003). Variability also differed by time since graduation for pain-related cases, with more experienced physicians showing greater variability (*p* = .020). No significant differences in variability were found based on patient involvement.

Turning to average differences (RQ3), a significant difference in mean estimated sick leave score was found between male and female physicians for pain-related conditions (*p* = .047), with male physicians recommending longer sick leave durations (*M* = 4.40, *SD* = 4.77) compared to female physicians (*M* = 3.08, *SD* = 1.95). A similar difference was found based on time since graduation (*p* = .016): physicians with more years of experience recommended longer sick leave durations for pain-related conditions (*M* = 4.28, *SD* = 4.41) compared to those with fewer years of experience (*M* = 2.92, *SD* = 1.80). No significant mean differences were found for mental health conditions, and no significant effects were observed for professional status or physicians’ perceptions of the degree of patient involvement in relation to the sick leave score.

### Associations with demographic and organisational factors

To address RQ4, correlations between the sick leave score and demographic or organisational factors were examined. Table 4 summarises the descriptive statistics (means and standard deviations) and intercorrelations between the sick leave score, physician characteristics, and perceived managerial support. A negative association was observed between perceived support from the frontline manager and score for mental health conditions, *r* = −.26, *p* = .002, as well as for pain-related sick leave, *r* = −.18, *p* = .030. Perceived support from the frontline manager was thus linked to a lower score for both mental health conditions and pain-related issues. There was a positive association between years of professional experience (years since graduation) and score for pain-related issues, *r* = .18, *p* = .034, indicating that the longer a physician had been licenced, the more extensively they tended to grant sick leave for patients with pain conditions. Additionally, a correlation was found between score for mental health conditions and score for pain, *r* = .50, *p* < .001, meaning that physicians who rendered higher scores did so regardless of the type of condition, and vice versa. This suggests a sick leave pattern among physicians that was independent of the type of condition.

**Table 4.** Mean, standard deviation and intercorrelations between the sick leave score, characteristics of the physicians and perceived work support.

Variable	Mean	*SD*	1	2	3	4
1. Patient involvement	5.65	0.97	–			
2. Frontline manager support	4.84	1.63	−.04	–		
3. Years since graduation	14.33	10.84	−.02	.06	–	
4. Sick leave score (mental health)	3.18	2.55	.01	−.26**	.07	–
5. Sick leave score (pain)	3.63	3.47	−.02	−.18*	.18*	.50***

* *p* < .05. **p < .01. ***p < .001.

## Discussion

The aim of this Swedish study was to examine the medical reliability and consistency of primary care physicians’ assessments of reduced work capacity, and to explore factors that may influence those assessments. The results showed substantial variation in the weighted sick leave measure (the sick leave score). Male physicians demonstrated greater variability and reported higher scores compared to female colleagues. Physicians with more years since graduation reported higher scores for pain-related conditions, while specialists showed less variability than non-specialists in mental health cases. Lower scores were associated with stronger perceived support from the frontline manager for both mental health and pain-related conditions. A relatively strong correlation between assessments of pain-related and mental health cases indicated that some physicians consistently assigned either higher or lower scores across conditions. Finally, no significant differences were found in relation to physicians’ reported ambition to involve patients in the sick leave process.

### Assessing reliability: How consistent are sick leave recommendations?

Perhaps the most striking finding was that it seems difficult to assess reduced work capacity in a consistent and legally secure manner. Physicians’ weighted assessments of reduced work capacity and the recommended duration of sick leave varied substantially across the participating physicians. Assessing work capacity is a task that many physicians in primary care find challenging [11], and there is a lack of clear guidelines on how to relate illness to work capacity in specific cases [3]. Moreover, training in insurance medicine is often inadequate [5,6].

The finding that assessments by male physicians differed from those of female physicians is particularly interesting given that previous studies have suggested the opposite: namely, that female physicians are more likely to recommend sick leave [13,14]. However, those studies typically examined sick leave as a binary outcome – whether it was recommended or not – whereas the present study assessed a weighted measure of the degree and length of the recommended leave. A notable feature of the present study’s design is that factors such as the treatment relationship and the patient’s attitude or influence during the consultation were not part of the assessment. This may partly account for the difference in findings and may hypothetically suggest that, in clinical practice, female physicians are more responsive to the patient’s behaviour and communication. Further research is needed to explore this in more depth. However, even though the patients were not present to influence the assessments in the present study, substantial variation in sick leave duration was still observed across the different patient cases. This suggests that, although the patient may be an important influencing factor, other aspects also play a significant role in shaping physicians’ assessments of sick leave duration, such as information about the patient’s work situation and demands. The assessment of work capacity needs to be understood as relational, shaped not only by the patient’s health but also by such contextual factors [22].

We also examined the potential impact on sick leave assessment of physicians’ reported ambitions to involve the patient in the sick leave process. However, no differences were found, either in terms of variability or in mean sick leave score. Physicians who reported a higher degree of patient involvement did not differ from those reporting lower levels of involvement. One possible explanation is that the overall ambition to involve the patient was relatively high across the sample, which may have reduced the potential to detect meaningful differences. Still, this result suggests a strong commitment to patient involvement, which is a key aspect of person-centred care – something strongly recommended in Swedish healthcare [32]. In the context of sick leave, working in a person-centred manner may include involving multiple professional roles based on the patient’s needs, making joint decisions with the patient regarding care and treatment, and recognising and utilising the patient’s own resources [3]. However, if the sick leave process becomes too strongly patient-driven, it may result in unnecessarily long sick leave periods [8,9,25]. In many cases, the patient can be seen as a predictor of the actual sick leave duration [33]. Physicians responsible for sickness certification must balance the patient’s expectations with the aim of working in a person-centred way, while also making an independent judgement about the appropriate duration of sick leave [20]. How this balance is managed may help to explain the observed variation in assessments of work capacity and sick leave duration [5]. In light of the high reported level of patient involvement, an important avenue for future research is to examine how primary care physicians define and apply the concept in practice.

The results showed different patterns, in terms of both variability and mean differences, depending on whether the cases concerned mental health conditions or pain-related issues. Two related physician characteristics – status as a specialist and time since graduation – showed almost opposite results. It is worth noting that not all physicians with long time since graduation were specialists. Physicians with *more years since graduation* showed significantly greater variability and reported higher sick leave scores for the pain-related conditions, whereas no such differences were observed for the mental health conditions. This may reflect changes in the medical education curriculum for those who graduated more recently, where there is now a stronger emphasis on the potential negative consequences of prolonged sick leave as an integral part of the medical school training programme. *Specialists*, on the other hand, showed significantly less variability than non-specialists, but only for the mental health cases. No significant differences in score were found for either of these groups. The lower variability among specialists may be explained by their greater *knowledge* and clinical experience, which they draw on when making complex assessments. This may result in more uniform recommendations for sick leave in cases involving mental health conditions. It is possible that mental health assessments are more difficult due to their reliance on subjective symptoms, making them harder to evaluate consistently compared to pain-related conditions [34]. Years of practice are typically associated with accumulated *experience* and the development of strategies for managing sick leave assessments. One might expect that such strategies would reduce variability, yet in this case, physicians with more years of experience showed greater variability for pain-related cases. One possible explanation is that local routines for managing these cases may vary, and when combined with individual strategies, this could result in more divergent assessments. Why this does not appear to affect assessments for mental health cases remains unclear and is something future research should further explore. The interpretation of these differences must also be considered in light of the fact that, as previously mentioned, the patients were not present as an influencing factor in the present study. In clinical settings, physicians with many years of experience may have developed strategies for managing unreasonable or demanding patient expectations. Conversely, physicians with less experience may be more susceptible to being influenced by patients’ wishes and may be more inclined to accommodate them in their assessments.

An interesting finding is the apparent presence of an individual approach to assessing reduced work capacity. That is, some physicians tended to assign higher sick leave scores regardless of the underlying condition, while others consistently assigned lower scores. This may reflect differences in the strategies physicians apply when working with medical certificates [8]. These strategies include taking either an active or a passive role in the assessment process. Bengtsson Boström et al. [8] found that physicians with more experience of failed rehabilitation cases were more likely to adopt a passive role. Understanding the underlying reasons for such assessment patterns is important, as unnecessarily long periods of sick leave may increase the risk of prolonged absence [13]. Patients who have been on sick leave for extended periods are also at greater risk of being granted permanent disability benefits and of increased mortality [28]. Despite these risks, it seems that few physicians discuss the potential consequences of prolonged sick leave with their patients [11].

Physicians who felt more supported by their frontline managers tended to assign lower sick leave scores. This could be understood in light of the fact that it is often difficult for physicians to take a firm stance and argue that a shorter period is more appropriate, particularly in relation to the patient and the potential pressure the physician may experience. Having strong managerial support may make it easier for physicians to act accordingly. Supporting this interpretation, previous research has shown that clear and well-established routines at the workplace reduce the risk of unnecessarily long sick leave periods [27]. Furthermore, since 2020, Swedish healthcare providers are obliged by law to offer rehabilitation coordination for patients on sick leave. This function is intended to support both the physician and the patient in the often complex sickness certification process. A recent interview study has shown that rehabilitation coordinators may relieve physicians by providing practical and emotional support, as well as increasing patients’ sense of security [35]. These findings highlight that physicians’ assessments do not occur in isolation but are influenced by the organisational context, including the resources available for rehabilitation coordination. Our results suggest that such contextual support may play an important role in how sick leave assessments are conducted, although more research is needed to examine the impact of rehabilitation coordinators on physicians’ decision-making.

### Practical implications

The findings of this study highlight the substantial variation in sick leave assessments for patients with mental health conditions and pain-related issues – even in the absence of patient influence. This underscores the importance of making the tacit knowledge behind these assessments more visible and placing greater emphasis on it. During data collection, informal discussions were sometimes initiated among the participating physicians after they had completed the questionnaire. These spontaneous conversations resembled workshops in which the cases were discussed and reflected upon. Such discussions could be formalised to create space for collective reflection and sharing of ideas on how to approach different types of situations. It is important that such reflective forums not only focus on the medical aspects of assessment but also address how physicians can manage patient interactions and navigate expectations and demands. Equally important is the opportunity for physicians to share experiences and knowledge about how best to involve patients in the process while also maintaining a professional balance and meeting the demands of the role. Person-centred care is actively promoted in Swedish healthcare policy [32], and any effort to strengthen this approach is likely to influence how patients perceive and experience the sick leave process.

Another key issue concerns how workplace factors influence the assessment process. In the present study, perceived support from one’s frontline manager was associated with lower sick leave scores. However, how this and other organisational factors influence or facilitate physicians’ work in practice remains an area in need of further research.

Greater awareness of both organisational and individual factors that influence sick leave assessments could be highly relevant for the training and continuing education of physicians – especially given the recurring calls for improved education in insurance medicine [5,6]. With such wide variation in sick leave recommendations, there is a potential risk that patients will not receive the support to which they are entitled, and that the sickness insurance system is applied inconsistently and unpredictably, contrary to the principle that ‘similar cases should be assessed in the same way’ [16, p.197]. In other words, there is a risk that the legal security of the assessment process is undermined [10], which may in turn complicate communication with the patient, who reasonably expects a coherent and fair recommendation regarding sick leave. This highlights the importance of focused training and continuing education for physicians in relation to the sick leave process.

### Strengths and limitations

The assessments of reduced work capacity and initial sick leave duration (in weeks) were based solely on the information provided in authentic medical certificates – diagnosis, functional impairment and activity limitations. As there was no interaction with an actual patient, this limits the extent to which the findings can be generalised to how physicians in primary care assess sick leave duration in clinical encounters with patients experiencing mental health conditions or pain-related issues. Ideally, all physicians would have assessed the same patient in person and under the same conditions – something that is, of course, not feasible in real-world clinical practice. However, the use of written certificates rather than clinical consultations can be considered a strength. The findings were based entirely on each physician’s independent judgement, unaffected by factors such as the treatment relationship or direct patient interaction. Given that the sick leave process is often described as patient-driven, and that this has been identified as a risk factor for the granting of unnecessarily long sick leave [8,9,25], this design makes it possible to more clearly isolate variations at the physician level.

Another strength is the use of real, anonymised medical certificates rather than constructed vignettes or fictional patient descriptions, which increases the ecological validity of the study. Additionally, all participating physicians assessed the same six cases, enabling direct comparisons between individual physicians. The sample was relatively large (*n* = 142) and included physicians from 16 healthcare centres across four counties, representing a mix of urban and rural contexts. This enhances the generalisability of the findings within the Swedish primary care system.

Nevertheless, several limitations should be noted. The study included only two diagnostic categories – mental health conditions and pain-related issues – which, while the two most common diagnostic entities, do not reflect the full range of medical cases that require sick leave certification. Furthermore, physicians’ responses regarding organisational support, patient involvement their own work situation were based on self-report. As such, there is a potential risk of socially desirable responding. For example, participants may have rated their level of patient involvement more favourably in order to present themselves in a more positive light [36]. This potential bias should be considered when interpreting the results.

It should also be noted that some of the correlations, although statistically significant, were relatively weak – for example, between support from the frontline manager and sick leave scores for mental health and pain-related issues and between years of experience and pain-related scores. This means that these variables explain only a small proportion of the variance. Nevertheless, in an organisational context, even weak associations may provide useful insights into the processes at work, although such findings should be interpreted with caution.

Another potential limitation that may have influenced the results is that the assessments depended on the quality of the medical certificates. The more detailed the certificate, the easier it is for an external party to make an accurate evaluation. Given the demands of a busy workday, it is conceivable that physicians may not have invested maximum effort into writing flawless certificates, but rather aimed to meet the minimum requirements for approval by the Social Insurance Agency. In clinical practice, physicians have the opportunity to communicate with case officers at the Social Insurance Agency to provide additional details if needed for the evaluation of a certificate. However, the certificates used in this study were selected to represent a typical sample, rather than ‘perfect’ or particularly ‘poor’ examples.

Finally, there is no meaningful way to estimate response rate other than that all the physicians who attended the meeting at which data collection took place also participated. However, there is also a potential risk of selection bias, as physicians who chose to take part may be more engaged or interested in the topic, which could influence the generalisability of the findings to the wider population of primary care physicians.

## Conclusions

This study highlights substantial variability in how primary care physicians assess the degree and duration of sick leave for patients with mental health conditions and pain-related issues. These findings suggest that assessments of reduced work capacity may not be entirely reliable, which in turn may undermine fair and consistent assessments of patients’ work capacity. Individual physician characteristics – such as gender, specialist status and years of experience – as well as perceived organisational support, were associated with differences in assessment patterns. The observed variability underscores the need for strengthened education in insurance medicine, clearer guidelines and greater emphasis on reflective practice and organisational support structures. Enhancing transparency in assessment strategies and creating formal opportunities for professional reflection could contribute to more uniform, equitable and patient-centred sick leave processes. Future research should explore how organisational environments and clinical interactions further shape physicians’ sick leave assessments in everyday practice.

## Data Availability

The data that support the findings of this study are available from the corresponding author, MF, upon reasonable request.

## References

[1] Eurostat. Employment and unemployment. European Union, Brussels, Belgium, 2021. [Cited 2025, Oct 27]. Available from: https://ec.europa.eu/eurostat/web/lfs

[2] Dutrieux J. Swedish sickness absence in a European perspective, 1995–2022 [Den svenska sjukfrånvaron i ett europeiskt perspektiv, 1995–2022]; Swedish Social Insurance Agency [report], Stockholm, Sweden. 2023. [Cited 2025, Oct 27]. Available from: https://www.forsakringskassan.se/download/18.2af5a1181888fbe751e1b2/1693378024724/den-svenska-sjukfranvaron-i-ett-europeiskt-perspektiv-1995-2022-arbetsrapport-2023-2.pdf

[3] The Swedish Government, SOU. The right conditions for sick leave: report from the investigation into the importance of the medical certificate in sickness benefit cases [Rätt förutsättningar för sjukskrivning: betänkande av Utredningen om läkarintygets betydelse i sjukpenningärenden] (3. 48); The Swedish Government Public Reports, 2023. [Cited 2025, Oct 27] Available from: https://www.regeringen.se/rattsliga-dokument/statens-offentliga-utredningar/2023/08/sou-202348/

[4] Alexanderson K, Azad A, Haque M, et al. Läkares erfarenheter av samverkan med Försäkringskassan - kvalitativa och kvantitativa analyser av enkätsvar år 2017 och jämförelser med tidigare år. Karolinska Institute [report]. Stockholm, Sweden; 2020. [Cited 2025, Oct 27]. Available from: https://ki.se/media/100054/download

[5] National Board of Health and Welfare. The healthcare sector’s work with sick leave and rehabilitation: a situational report based on surveys with doctors and regional leadership in autumn 2022 [Hälso- och sjukvårdens arbete med sjukskrivning och rehabilitering. En lägesbeskrivning baserad på enkäter till läkare och regionledning hösten 2022]; 2023. Available from: https://www.socialstyrelsen.se/globalassets/sharepoint-dokument/artikelkatalog/ovrigt/2023-6-8595.pdf

[6] Nordling P, Priebe G, Björkelund C, et al. Assessing work capacity - reviewing the what and how of physicians’ clinical practice. BMC Fam Pract. 2020;21(1):72. doi: 10.1186/s12875-020-01134-9. 32340611 PMC7187489

[7] Alexanderson K, Arrelöv B, Friberg E, et al. Läkares erfarenheter av arbete med sjukskrivning av patienter Resultat från en enkät år 2017 och jämförelser med resultat från motsvarande enkäter år 2012, 2008 respektive 2004. Karolinska Institute [report], Stockholm, Sweden: (ISBN 978-91-981256-0-3). 2018.

[8] Bengtsson Boström K, Starzmann K, Östberg A-L. Primary care physicians’ concerned voices on sickness certification after a period of reorganization. Focus group interviews in Sweden. Scand J Prim Health Care. 2020;38(2):146–155. doi: 10.1080/02813432.2020.1753341. 32314635 PMC8570729

[9] Engblom M, Nilsson G, Arrelöv B, et al. Frequency and severity of problems that general practitioners experience regarding sickness certification. Scand J Prim Health Care. 2011;29(4):227–233. doi: 10.3109/02813432.2011.628235. 22126222 PMC3308465

[10] Swedish National Audit Office. Assessment of work ability in mental health issues [Bedömning av arbetsförmåga vid psykisk ohälsa. RIR. 2018;2018:11.

[11] Bertilsson M, Maeland S, Löve J, et al. The capacity to work puzzle: a qualitative study of physicians’ assessments for patients with common mental disorders. BMC Fam Pract. 2018;19(1):133. doi: 10.1186/s12875-018-0815-5. 30060734 PMC6066915

[12] Wainwright E, Wainwright D, Keogh E, et al. The social negotiation of fitness for work: tensions in doctor-patient relationships over medical certification of chronic pain. Health. 2015;19(1):17–33. doi: 10.1177/1363459314530738. 24821926

[13] Englund L, Svardsudd K. Sick-listing habits among general practitioners in a Swedish county. Scand J Prim Health Care. 2000;18(2):81–86. doi: 10.1080/028134300750018954. 10944061

[14] Englund L, Tibblin G, Svärdsudd K. Variations in sick-listing practice among male and female physicians of different specialities based on case vignettes. Scand J Prim Health Care. 2000;18(1):48–52. doi: 10.1080/02813430050202569. 10811044

[15] Nilsen S, Werner EL, Maeland S, et al. Considerations made by the general practitioner when dealing with sick-listing of patients suffering from subjective and composite health complaints. Scand J Prim Health Care. 2011;29(1):7–12. doi: 10.3109/02813432.2010.514191. 20822375 PMC3347936

[16] Ministry of Health and Social Affairs, SOU. 2024. An evaluation of changes in the sickness insurance regulations during 2021 and 2022 [En Utvärdering Av Förändringar i Sjukförsäkringens Regelvärk under 2021 Och 2022]. Ministry of Health and Social Affairs [report]; 2024 (26). Available from: https://www.regeringen.se/rattsliga-dokument/statens-offentliga-utredningar/2024/04/sou-202426/

[17] Maeland S, Werner EL, Rosendal M, et al. Sick-leave decisions for patients with severe subjective health complaints presenting in primary care: a cross-sectional study in Norway, Sweden, and Denmark. Scand J Prim Health Care. 2013;31(4):227–234. doi: 10.3109/02813432.2013.844412. 24164371 PMC3860299

[18] Slebus FG, Kuijer PFM, Willems JHBM, et al. Work ability assessment in prolonged depressive illness. Occup Med. 2010;60(4):307–309. doi: 10.1093/occmed/kqq079. 20511270

[19] Werner EL, Merkus SL, Mæland S, et al. Physicians’ assessments of work capacity in patients with severe subjective health complaints: a cross-sectional study on differences between five European countries. BMJ Open,. 2016;6(7):e011316. doi: 10.1136/bmjopen-2016-011316. PMC4947783 27417198

[20] Letrilliart L, Barrau A. Difficulties with the sickness certification process in general practice and possible solutions: a systematic review. Eur J Gen Pract. 2012;18(4):219–228. doi: 10.3109/13814788.2012.727795. 23205966

[21] Brenninkmeijer V, Houtman I, Blonk R. Depressed and absent from work: predicting prolonged depressive symptomatology among employees. Occup Med. 2008;58(4):295–301. doi: 10.1093/occmed/kqn043. 18434294

[22] Hellman T, Wåhlin C. Företagshälsans guide om arbetsförmåga - begrepp, samtal och utredning. Sveriges Företagshälsor, Stockholm, Sweden; 2021 [ISBN 978-91-519-5201-7]. [Cited 2025, Oct 27]. Available from: https://www.foretagshalsor.se/sites/default/files/2021-11/Arbetsförmågeguide_2021.pdf

[23] Nygård C-H, Rantanen T. The need for methods to measure capacity and incapacity from working life to old age. Occup Environ Med. 2017;74(7):467–467. doi: 10.1136/oemed-2017-104291. 28468930

[24] Löfgren A, Hagberg J, Arrelöv B, et al. Frequency and nature of problems associated with sickness certification tasks: a cross-sectional questionnaire study of 5455 physicians. Scand J Prim Health Care. 2007;25(3):178–185. doi: 10.1080/02813430701430854. 17846937 PMC3379778

[25] Nilsson GH, Arrelöv B, Lindholm C, et al. Psychiatrists’ work with sickness certification: frequency, experiences and severity of the certification tasks in a national survey in Sweden. BMC Health Serv Res. 2012;12(1):362. doi: 10.1186/1472-6963-12-362. 23075202 PMC3480832

[26] Arrelöv B, Alexanderson K, Hagberg J, et al. Dealing with sickness certification - a survey of problems and strategies among general practitioners and orthopaedic surgeons. BMC Public Health. 2007;7(1):273. doi: 10.1186/1471-2458-7-273. 17910746 PMC2089078

[27] Bränstrom R, Arrelov B, Gustavsson C, et al. Reasons for and factors associated with issuing sickness certificates for longer periods than necessary: results from a nationwide survey of physicians. BMC Public Health,. 2013;13:478. doi: 10.1186/1471-2458-13-478. 23679866 PMC3691717

[28] Mittendorfer Rutz E, Kjeldgård L, Wikman A, et al. Sjukskrivning i psykiska diagnoser och risk för att få sjuk- eller aktivitetsersättning eller för förtida död. Swedish Social Insurance Agency, Stockholm, Sweden; [webb page]; 2011. [Cited 2025, 27 Oct] Available from: https://ki.se/cns/forskning/forsakringsmedicin/forskargrupper-vid-forsakringsmedicin/kvinnors-och-mans-sjukfranvaro

[29] Swedish Social Insurance Agency. Insurance medicine at the Swedish Social Insurance Agency [Försäkringsmedicin på Försäkringskassan]; 2023. Available from: https://www.forsakringskassan.se/halso-och-sjukvarden/forsakringsmedicin/forsakringsmedicin-pa-forsakringskassan

[30] Swedish Social Insurance Agency. Certificate for sickness benefit [Intyg för sjukpenning]; 2023. Swedish Social Insurance Agency, Stockholm, Sweden; [webb page]. Available from: https://www.forsakringskassan.se/halso-och-sjukvarden/sjukdom-och-skada/intyg-for-sjukpenning

[31] Swedish Social Insurance Agency. Social insurance in figures 2023 [Socialförsäkringen i siffror 2023]; Swedish Social Insurance Agency [report], Stockholm, Sweden (ISBN: 978-91-7500-406-8), 2023. [Cited 2025, Oct 27]. Available from: https://www.forsakringskassan.se/download/18.2af5a1181888fbe751eaf/1687877749315/socialforsakringen-i-siffror-2023.pdf

[32] Swedish Parliament. The Patient Act [Patientlagen] (2014:821). 2014. Ministry of Health and Social Affairs, Stockholm, Sweden, 2014. [Cited 2025, Oct 27]. Available from: https://www.riksdagen.se/sv/dokument-och-lagar/dokument/svensk-forfattningssamling/patientlag-2014821_sfs-2014-821/

[33] Ståhl C, Karlsson N, Gerdle B, et al. Predictive validity of general work ability assessments in the context of sickness insurance. J Rehabil Med. 2021;53(4):jrm00177. doi: 10.2340/16501977-2798. 33594444 PMC8814851

[34] Nilsing E, Söderberg E, Berterö C, et al. Primary healthcare professionals’ experiences of the sick leave process: a focus group study in Sweden. J Occup Rehabil. 2013;23(3):450–461. doi: 10.1007/s10926-013-9418-0. 23345119

[35] Sällstrom Randsalu L, Stigmar K. No longer alone. Scand J Prim Health Care. 2025;43(3):602–612. doi: 10.1080/02813432.2025.2486145. 40183587 PMC12377134

[36] Podsakoff PM, Organ DW. Self-reports in organizational research: problems and prospects. J Manag. 1986;12(4):531–544. doi: 10.1177/014920638601200408.

